# Recent Advances in the Synthesis of 3,n-Fused Tricyclic Indole Skeletons via Palladium-Catalyzed Domino Reactions

**DOI:** 10.3390/molecules28041647

**Published:** 2023-02-08

**Authors:** Liangxin Fan, Xinxin Zhu, Xingyuan Liu, Fangyu He, Guoyu Yang, Cuilian Xu, Xifa Yang

**Affiliations:** 1 Department of Chemical Biology, School of Sciences, Henan Agricultural University, Zhengzhou 450002, China; 2 Institute of Pesticide, School of Plant Protection, Henan Agricultural University, Zhengzhou 450002, China

**Keywords:** palladium, tricyclic indoles, domino reaction, catalyst, total synthesis

## Abstract

3,n-fused (*n* = 4–7) tricyclic indoles are pervasive motifs, embedded in a variety of biologically active molecules and natural products. Thus, numerous catalytic methods have been developed for the synthesis of these skeletons over the past few decades. In particular, palladium-catalyzed transformations have received much attention in recent years. This review summarizes recent developments in the synthesis of these tricyclic indoles with palladium-catalyzed domino reactions and their applications in the total synthesis of representative natural products.

## 1. Introduction

**Scheme 1:**
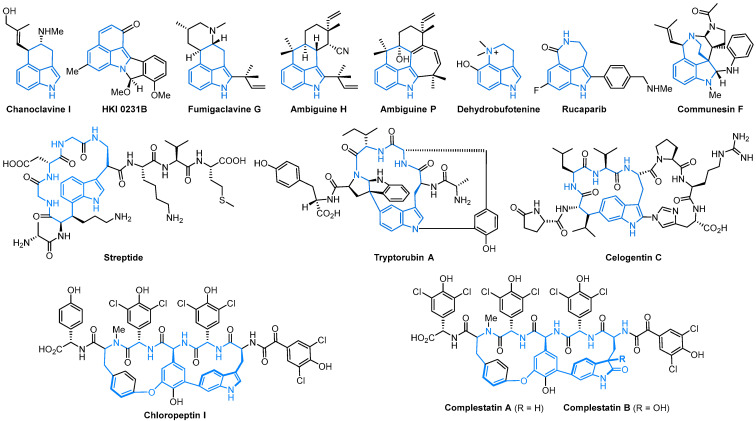
Representative examples of biologically active molecules containing tricyclic indole cores.

Indole is one of the most significant nitrogen heterocycles, owing to its unique biological activity and wide existence in numerous natural products, bioactive molecules, pharmaceuticals, and functional materials [1,2,3,4,5,6]. Among the numerous indole alkaloids, 3,n-fused (*n* = 4–7) tricyclic indoles have attracted much attention for their typical biological activities and synthetic challenges in recent years [7,8]. The tricyclic indole nucleus is found in a myriad of natural products and pharmaceutical molecules (Scheme 1), such as HKI 0231B [9], dehydrobufotenine [10], ambiguine H [11], tryptorubin A [12], celogentin C [13], chloropeptin I [14], and streptide [15]. Accordingly, a number of strategies have been established for the synthesis of these skeletons, including intramolecular Fischer indole synthesis, Witkop photocyclizations, Diels-Alder reactions, Friedel-Crafts reactions, Pictet-Spengler reactions, 6π-electrocyclizations, and transition-metal-catalyzed domino reactions.

In recent years, numerous advances have been achieved via transition-metal-catalyzed domino reaction. Among them, palladium has been widely used as a versatile catalyst for their typical atom orbital to get a series of tricyclic indole skeletons. The seminal work on the synthesis of tryptophan derivatives was reported by Roberts in 1994 [16]. In this work, the palladium catalyst was employed to complete the bridge between C3 and C4. After that, especially within the last ten years, a variety of protocols for synthesis of these molecules have been developed and refined by researchers. Moreover, the palladium catalyst was also used extensively in cross-coupling reactions, such as Buchwald-Hartwig, Suzuki-Miyaura, Heck, Sonogashira, Negishi, and Stille to synthesize a variety of useful compounds [17,18].

Over the past few years, some reviews on the synthesis of 3,n-fused tricyclic indoles were documented [7,8]. However, to the best of our knowledge, there is no review that summarizes the recent progress for the synthesis of tricyclic indoles via transition metal catalyst. Herein, we want to provide a short review mainly focused on recent advances in the synthesis of tricyclic indoles via the palladium-catalyzed domino reaction up to 2023. Furthermore, the total synthesis of representative natural products obtained by the palladium-catalyzed domino reaction is also described in this paper.

## 2. Intramolecular Cyclization

**Scheme 2:**
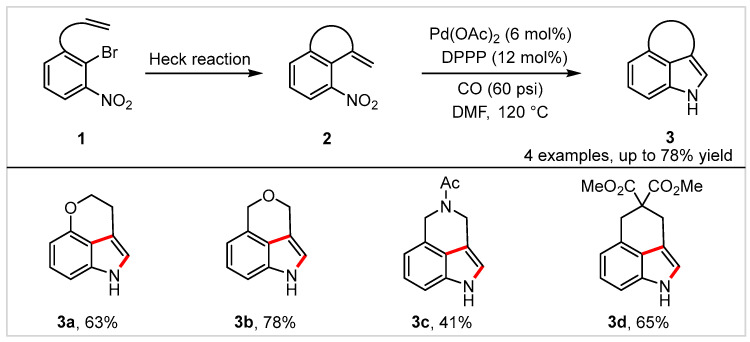
Synthesis of 3,4-fused tricyclic indoles via two consecutive palladium-catalyzed reactions.

The Heck reaction [19] is one of the most powerful methods for generating new C-C bonds, and has been used in numerous syntheses of high value-added chemicals and complex molecules. In 1999, Söderberg and co-workers [20] successfully developed a practical method for synthesizing 3,4-fused tricyclic indole skeletons via two consecutive palladium-catalyzed reactions, using an intramolecular Heck reaction followed by reductive *N*-heteroannulation. The reaction afforded various tricyclic indole products (**3a**–**3d**) in 41–78% yields with 5 mol% Pd(OAc)_2_ as catalyst, 12 mol% 1,3-bis(diphenylphosphino)propane (DPPP) as ligand, 60 psi carbon monoxide as reductant, and polar DMF as solvent (Scheme 2). Notably, compared to the previous Heck reaction, the relative rate of alkene insertion in this reaction is higher, likely due to the presence of the strong electron-withdrawing nitro group. Six years later, the same group [21] reported a similar method for preparation of 3,4-fused tricyclic indoles with 6–8-membered rings. With respect to the scope of substrates, nitrogen- and oxygen-containing rings work well in this reaction as well, except for the carbocyclic ring.

**Scheme 3:**
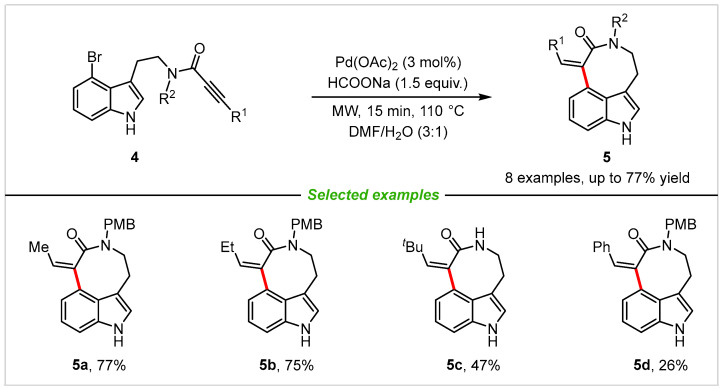
Synthesis of 3,4-fused tricyclic indoles through palladium-catalyzed intramolecular acetylene hydroarylation.

Medium-sized ring fused tricyclic indoles are quite useful structures in numerous natural products such as decursivine and serotobenine. In 2011, Van der Eycken and co-workers [22] reported a novel method for the construction of amide type of 3,4-fused tricyclic indole derivatives through a palladium-catalyzed intramolecular acetylene hydroarylation reaction with excellent regio- and stereoselectivity (Scheme 3). In this report, various nitrogen protecting groups and alkyne substituents, such as methyl (**5a**), ethyl (**5b**), *tert*-butyl (**5c**), phenyl (**5d**), and silyl groups were successfully tested and tolerated well, delivering the target products in moderate to good yields, and the structures were confirmed by ^1^H NMR, ^13^C NMR spectroscopy, HRMS (EI), and X-ray crystallography. The reaction is rapid, mild, regioselective, stereoselective and proceeds with good yields. It is worth noting that substrates bearing a free NH group (**5c**) also reacted smoothly in this reaction.

**Scheme 4:**
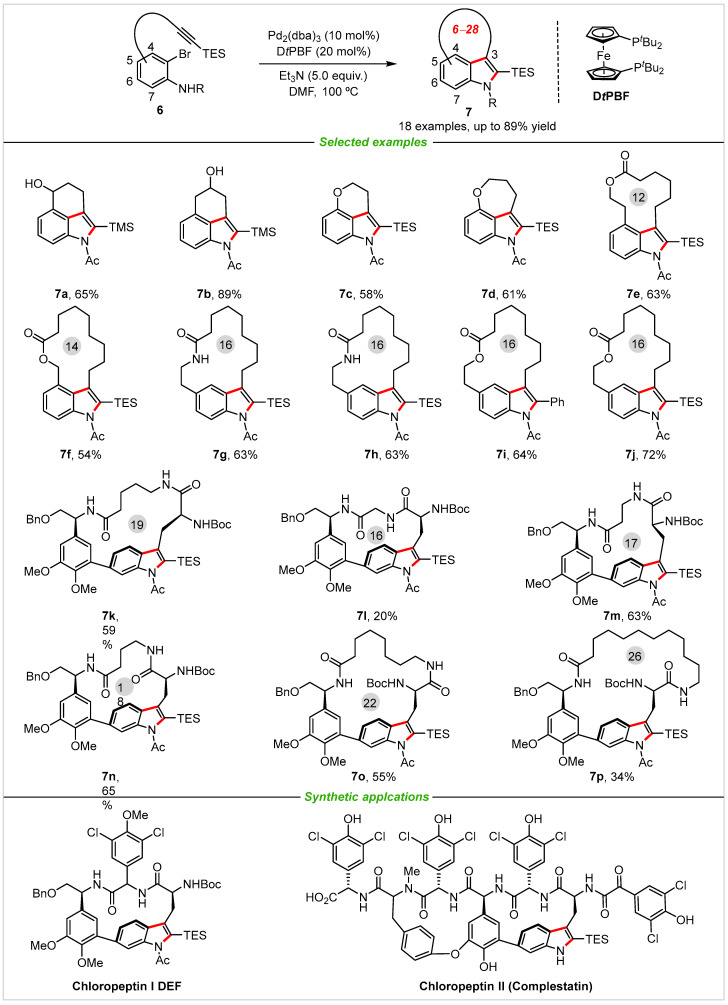
Synthesis of diversely functionalized 3,4-, 3,5-, and 3,6-fused tricyclic indoles **7** with the alkyne tethered *ortho*-bromoanilines.

As we know, Larock indole synthesis is one of the most efficient methods for the rapid construction of indole skeletons from halo-anilines and alkynes with palladium catalyst [23,24]. In 2013, Boger and co-workers [25] successfully reported the first example for rapid assembly of 3,(4-6)-tricyclic indoles **7** via intramolecular Larock indole annulation with a powerful Pd_2_(dba)_3_/D*t*PBF catalyst system, and catalyst and other reaction parameters were examined in detail (Scheme 4). This novel transformation features excellent functional-group tolerance (18 examples), good yields (up to 89% yield), and provides an efficient strategy to afford natural product chloropeptin I, chloropeptin II DEF ring system and key isomers directly. The ring size ranges from 6 to 28, which includes peptide chain and conventional carbon chain. More importantly, the TES or TMS group are easily removed from the molecules, leading to the formation of various indoline skeletons. It is worth noting that the established method is a good complement to the Stille or Suzuki cross-coupling reactions for the synthesis of cyclic or macrocyclic ring indole systems [26,27].

**Scheme 5:**
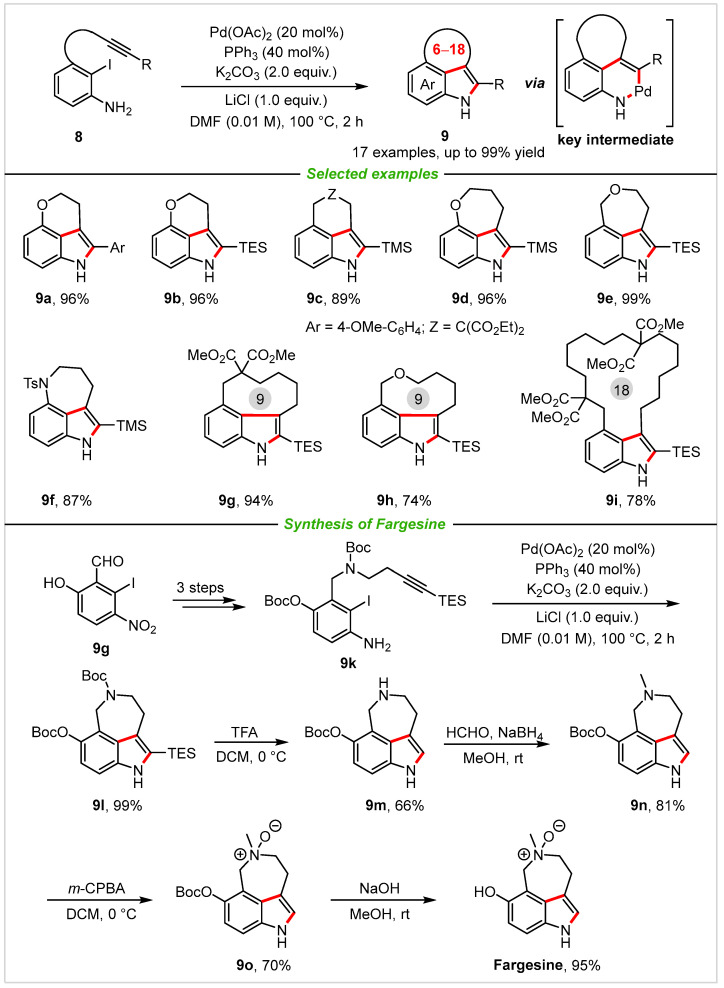
Synthesis of 3,4-fused tricyclic indoles via palladium-catalyzed intramolecular annulation of internal alkyne tethered *ortho*-iodoanilines and total synthesis of fargesine.

In the same year, Jia and co-workers [28] successfully disclosed a new and general method for the synthesis of 3,4-fused tricyclic indole derivatives through intramolecular Larock indole annulation from internal alkyne tethered *ortho*-iodoaniline derivatives, and these skeletons are often embedded in numerous natural products and bioactive molecules (Scheme 5). After extensive condition studies, the optimal reaction conditions are as follows: Pd(OAc)_2_ (20 mol%), PPh_3_ (40 mol%), K_2_CO_3_ (2.0 equiv.), and LiCl (1.0 equiv.) in DMF at 100 °C under nitrogen atmosphere. Applying the Pd/PPh_3_ catalyst system to the indole-synthesized reaction successfully produced 6–18-membered ring fused tricyclic indoles **9** in good to excellent yields (up to 99% yield), and various linkers, including carbon- (**9c**,**9g**,**9i**)), oxygen- (**9a**,**9b**,**9d**,**9e**,**9h**), or nitrogen- (**9f**) are all tolerated well. In addition, the method was highlighted by the total synthesis of the natural product fargesine, which was isolated from the root and stem of *Evodia fargesii* Dode. Notably, the authors also explored the reaction with the easily accessible *ortho*-bromoaniline-type substrates. Considering the lower reactivity of the C-Br bond, some representative ligands were tested to complete the ideal conversion, results showed that electron rich Me-phos or DPPP gave the best yields. Later, the method was also extended to the synthesis of 3,5-fused tricyclic indole scaffolds by the same group [29], giving a variety of 3,5-fused macrocyclic with good yields, and the method was also used to afford the tetrahydropyrrolo [4,3,2-*de*]quinolone ring.

**Scheme 6:**
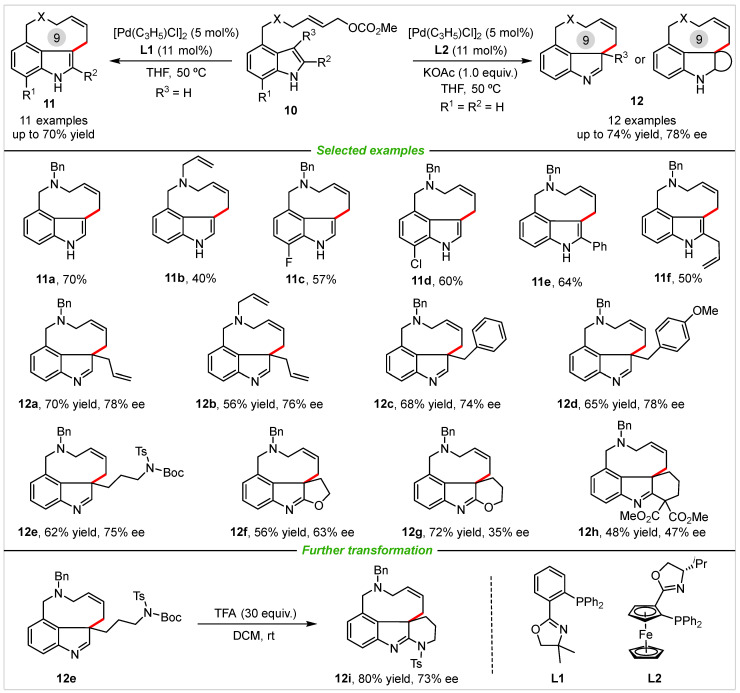
Diversity oriented synthesis of 3,4-fused tricyclic indole and their derivatives via palladium-catalyzed allylic alkylation reactions.

The strategy of diversity oriented synthesis (DOS) is of great importance in organic synthesis, medicine chemistry, agrochemical chemistry, and material sciences, which aims to efficient collections of small molecules with diverse appendages, functional groups, stereochemistry, and skeletons [30]. In 2013, the You group [31] developed an efficient, Pd(0)-catalyzed allylic alkylation protocol for the synthesis of 3,4-fused tricyclic indole derivatives from readily available 3-subsituted indoles at 50 °C (Scheme 6). The phosphine ligand was crucial for this Pd-catalyzed allylic alkylation reaction, results showed that the 2-(2-(diphenylphosphanyl)phenyl)-4,4-dimethyl-4,5-dihydrooxazole (**L1**) proved to be the best ligand and led to the formation of tricyclic indole product (**11a**) in 70% yield. Substituents at the nitrogen such as benzyl and allyl could be well-tolerated, and delivered tricyclic indoles in moderate yields. Indole substrates substituted with fluorine (**11c**) and chlorine (**11d**) at the 7-position generated the products with 57% and 60% yield, respectively. Moreover, various 3-substituted substrates were used to examine the substrate scope of the Pd-catalyzed allylic dearomatization reaction under slightly modified conditions. In the transformation, screening of various chiral ligands showed that planar chiral ferrocene-based ligand (**L2**) gave the highest value of ee. Substrates bearing methyl, allyl, benzyl, and various nucleophilic substituents at the 3-position of indole could be applied in the reaction and gave the desired products in good yields with moderate enantioselectivity (**12a**–**12h**). Notably, when an excess of trifluoroacetic acid (TFA) was added to (**12e**) at room temperature, tetracyclic product (**12i**) was achieved with 80% yield and 73% ee via deprotection of the Boc group followed by addition to imine.

**Scheme 7:**
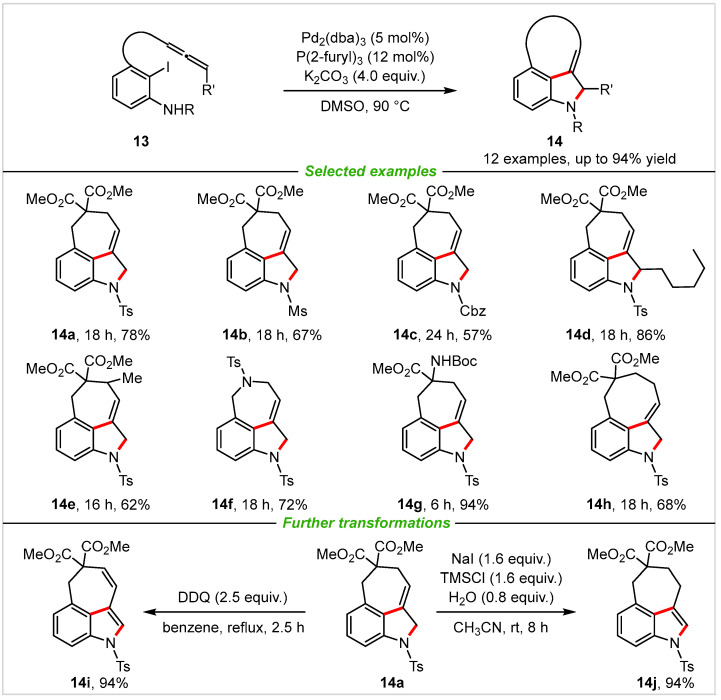
Palladium-catalyzed intramolecular annulation of allene tethered *ortho*-iodoanilines.

As important raw materials, allenes, which possess unique reactivity compared to alkenes, alkynes, and others, owing to the presence of two cumulative orthogonal π-bonds [32,33,34,35,36], have proven to be key synthetic intermediates in construction of various organic molecules. In particular, allenes generally react with aryl halide to produce π-allylpalladium(II) species in the presence of Pd(0) catalyst through a classic Heck insertion process. In 2015, Nemoto and co-workers [37] successfully demonstrated a novel Pd-catalyzed cascade cyclization with allene tethered *ortho*-iodoanilines **13** as starting materials. This enabled rapidly assembled 3,4-fused tricyclic 3-alkylidene indoline scaffolds through a sequence of oxidative addition, allene insertion, and allylic amination, thus yielding a new class of 3,4-tricyclic 3-alkylidene indoline skeletons in good to excellent yields with broad substrates scope (Scheme 7). Furthermore, the 3,4-tricyclic 3-alkylidene indolines were divergently transformed into three types of 3,4-fused tricyclic indoles in 94% to quantitative yield with rather simple operation, successfully demonstrating the utility of this cascade process. Alternatively, the desired 3,4-fused tricyclic indoles could also be obtained by oxidation of products **14** with DDQ or PCC.

**Scheme 8:**
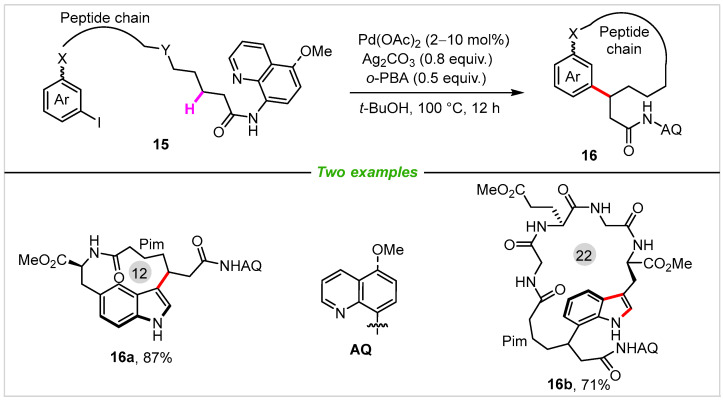
Synthesis of peptide-based macrocycles via palladium-catalyzed intramolecular C(sp^3^)-H arylation.

In many macrocyclic natural products, peptide-based macrocycles are pervasive motifs in medicine molecules [38,39,40]. Among them, drugs, such as anticancer agent octreotide, antibiotic vancomycin, and immunosuppressant cyclosporin are typical molecules. Therefore, a concise method for the synthesis of these skeletons is highly attractive and challenging. In 2018, Chen, Liu, Shen, Qi, He, and co-workers [41] described an amide and pyridine directed β-C(sp^3^)-H arylation reaction to access diverse peptide macrocycles under Pd(II) catalysis (Scheme 8). In this transformation, 3,5-fused tricyclic indole (**16a**) and 3,7-fused tricyclic indole (**16b**) were obtained in 87% and 71% yields, respectively.

## 3. Intermolecular Cyclization

**Scheme 9:**
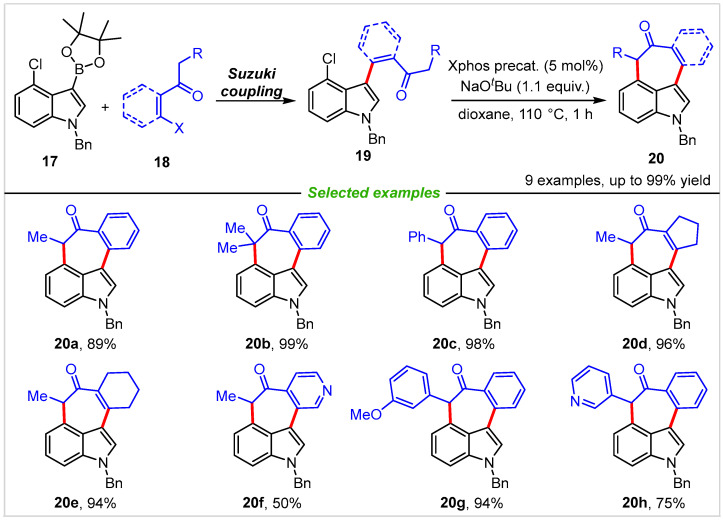
Synthesis of tetracyclic indoles via Suzuki reaction and intramolecular α-arylation of ketones.

Construction of new C-C bonds by palladium-catalyzed α-arylation of ketones has emerged as an attractive method in recent years [42]. To date, numerous efforts have been devoted to develop this methodology, and various functionalized molecules have been synthesized. However, application of this method for the synthesis of tetracyclic indoles has not been achieved so far. In 2012, Cuny and co-workers [43] reported a straightforward synthesis of tetracyclic indoles via intramolecular α-arylation of ketones using monoligated Pd(0) catalyst (Scheme 9). The choice of catalyst was vital to access the desired tetracyclic indole product in good to excellent yield. In the presence of the Pd-complex the functionalized het(aryl)ketones transformed to the desired products in 50–99% yields (methyl **20a** and **20f**, gem-dimethyl **20b**, phenyl **20c**, 3-methoxyphenyl **20g**, and pyridyl **20h**). Moreover, both the cyclopentenyl and cyclohexenyl substrates were tolerated well, giving **20d**, **20e** in a 96% and 94% yield, respectively. It is worth mentioning that the Pd catalytic system features good functional-group tolerance, good to excellent yields, low catalyst loading, and short reaction times. Furthermore, double α-arylation of ketones by one-pot procedures were also presented.

**Scheme 10:**
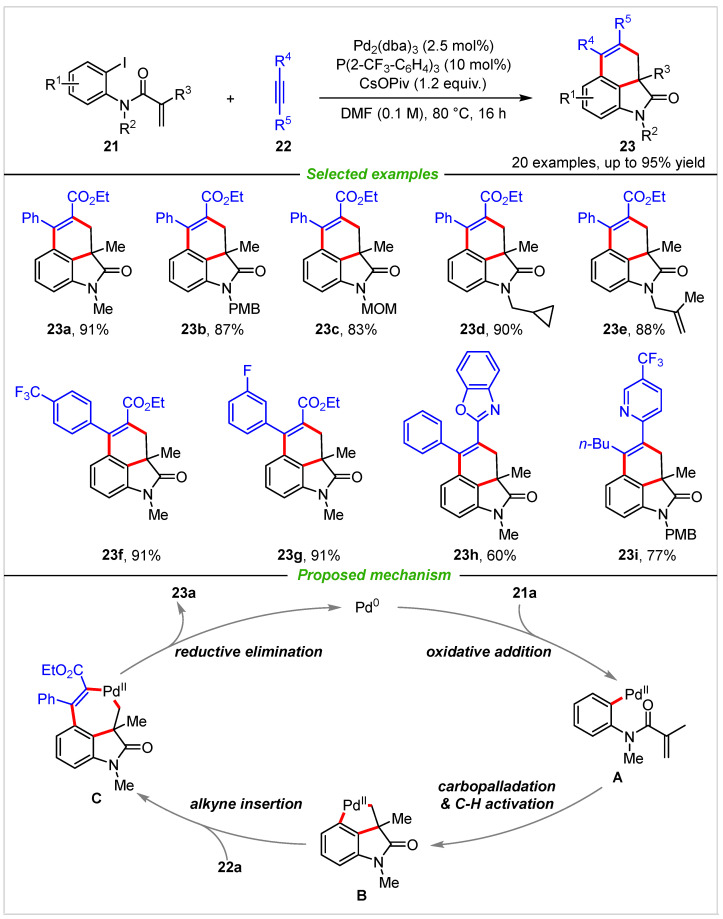
Synthesis of 3,4-fused tricyclic indolones via palladium-catalyzed cascade annulation between *ortho*-iodoacrylamides **21** and unsymmetrical internal alkynes **22**.

In 2018, based on their previous work and others, Lautens and co-workers [44] successfully discovered a novel Pd_2_(dba)_3_/P(2-CF_3_-C_6_H_4_)_3_-catalyzed domino reaction to give 3,4-fused tricyclic indolones in good to high yields and excellent regioselectivities with *ortho*-iodoacrylamides **21** and internal alkynes **22** via intramolecular carbopalladation/*ortho* C-H activation/alkyne insertion sequence (Scheme 10). Various coupling partners were investigated and tolerated well. Specifically, the acrylamide substrates containing *N*-, α-, and aryl-substituents, and several unsymmetrical alkynes were subjected to the reaction conditions and the corresponding reaction products were isolated as single regioisomers (**23a**–**23i**). Based on previous reports and experiment results, a plausible mechanism via a Pd(0)/Pd(II) catalytic cycle is depicted in the bottom of Scheme 10. Initially, acrylamide substrate (**21a**) undergoes oxidative addition to give aryl palladium species **A**. Next, intramolecular carbopalladation of alkene moiety followed by C(sp^2^)-H activation led to a five-membered palladacycle **B**. Afterward, coordination and migratory insertion of alkyne (**22a**) to five-membered palladacycle **B** furnished the seven-membered palladacycle **C.** Finally, reductive elimination of the intermediate C releases desired product (**23a**) and regenerates an active Pd(0) catalyst.

**Scheme 11:**
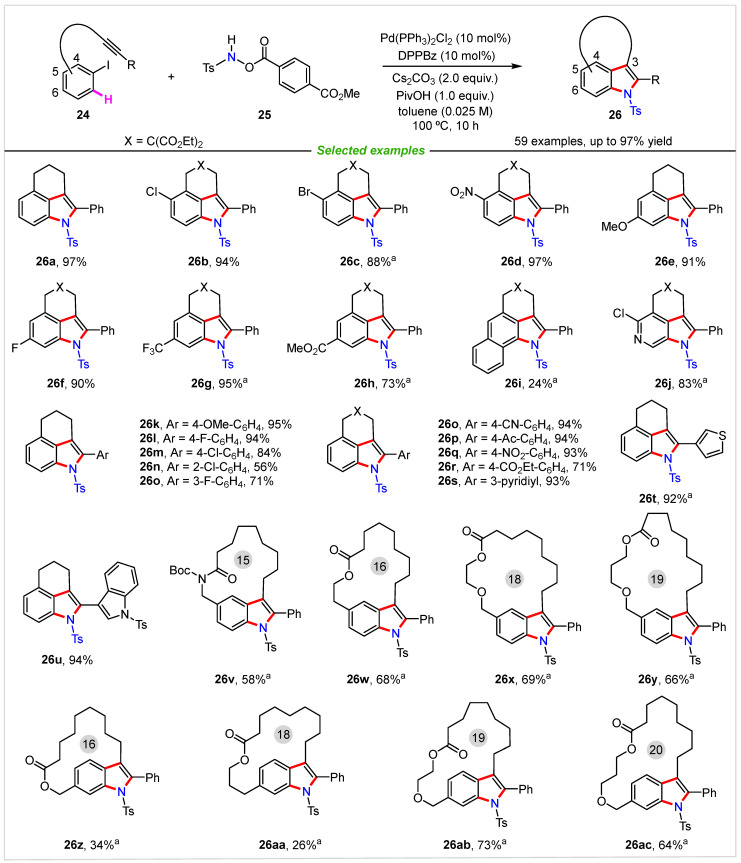
Palladium-catalyzed [2 + 2 + 1] annulation with internal alkyne tethered aryl iodide **24** and secondary hydroxylamines **25**. ^a^ 120 °C was used.

In 2020, Luan and co-workers [45] elegantly developed the first C(sp^2^)-H amination of alkyne-tethered aryl iodides for the rapid preparation of a series of tricyclic indoles using secondary hydroxylamines as the bifunctional single-nitrogen source under mild reaction conditions (Scheme 11). Initially, though various *N*-substituted secondary hydroxylamines were systematically evaluated, only Ts-NH-OBz could be promoted for the titled reaction in 8% yield. Next, various benzoyl *O*-substituents of amino agents, including steric effect and electron effect, have been examined in this transformation. In particular, substrates containing an ester in *para*-position led to the formation of tricyclic indole (**26a**) in 97% yield. Notably, the role of PivOH was crucial in obtaining the tricyclic indole products, which may be due to its ability to facilitate the process of C-H activation. For the scope of 3,4-fused tricyclic indoles, various aryl iodides, linkers, and alkyne termini were explored with good to excellent yields (**26b**–**26u**). Notably, the pyridyl and thienyl group, which might induce strong coordination toward the Pd(II) species, were found to proceed smoothly to give corresponding tricyclic indole products (**26s**, **26t**) in 93% and 92% yields, respectively. More importantly, the reaction works well to afford 3,4-fused tricyclic indoles as well as 3,5-, and 3,6-fused tricyclic indoles (**26v**–**26ac**) in acceptable yields. Of note, these tricyclic indoles have been observed as the key core for a range of biologically active natural and unnatural products [12,46,47].

**Scheme 12:**
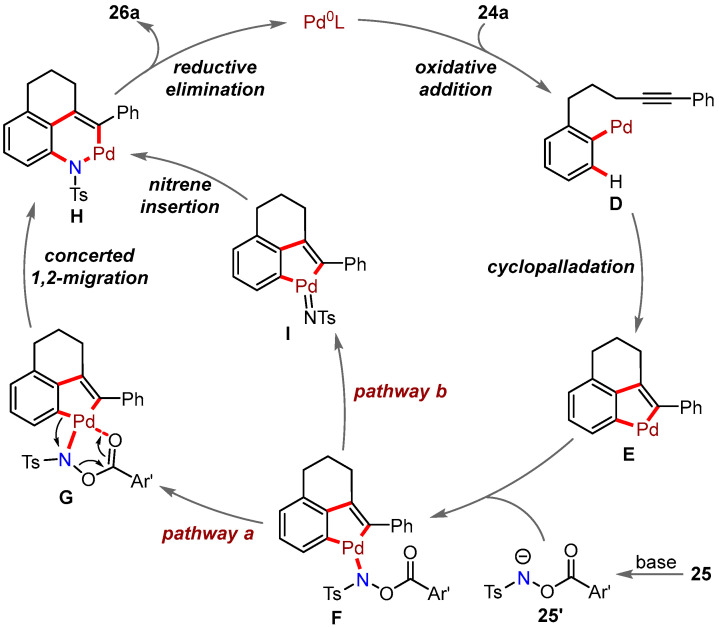
Proposed mechanism of Pd(0)-catalyzed [2 + 2 + 1] annulation.

On the basis of previous literature reports and detailed mechanistic investigations [48,49,50], the authors proposed a plausible pathway as depicted in Scheme 12. The reaction initiated with generation of aryl palladium species **D** via oxidative addition of iodobenzene moiety of **24a** to Pd(0). Next, intramolecular carbopalladation of alkynes followed by C-H activation led to the formation of five-membered palladacycle **E**. After being deprotonated with Cs_2_CO_3_, the amino agent **25′** coordinates with electrophilic Pd(II) species **E** and undergoes concerted 1,2-migration insertion of the aryls to afford the six-membered palladacycle **H**. Finally, reductive elimination of the intermediate H provided the desired tricyclic indole product **26a** with the concomitant regeneration of the active Pd(0) catalyst. Alternatively, formation of formal nitrene intermediate **I** is followed by nitrene insertion to furnish the six-membered palladacycle **H**. Intermediate **H** undergoes reductive elimination to yield the desired product **26a** and regenerate an active Pd(0) catalyst.

**Scheme 13:**
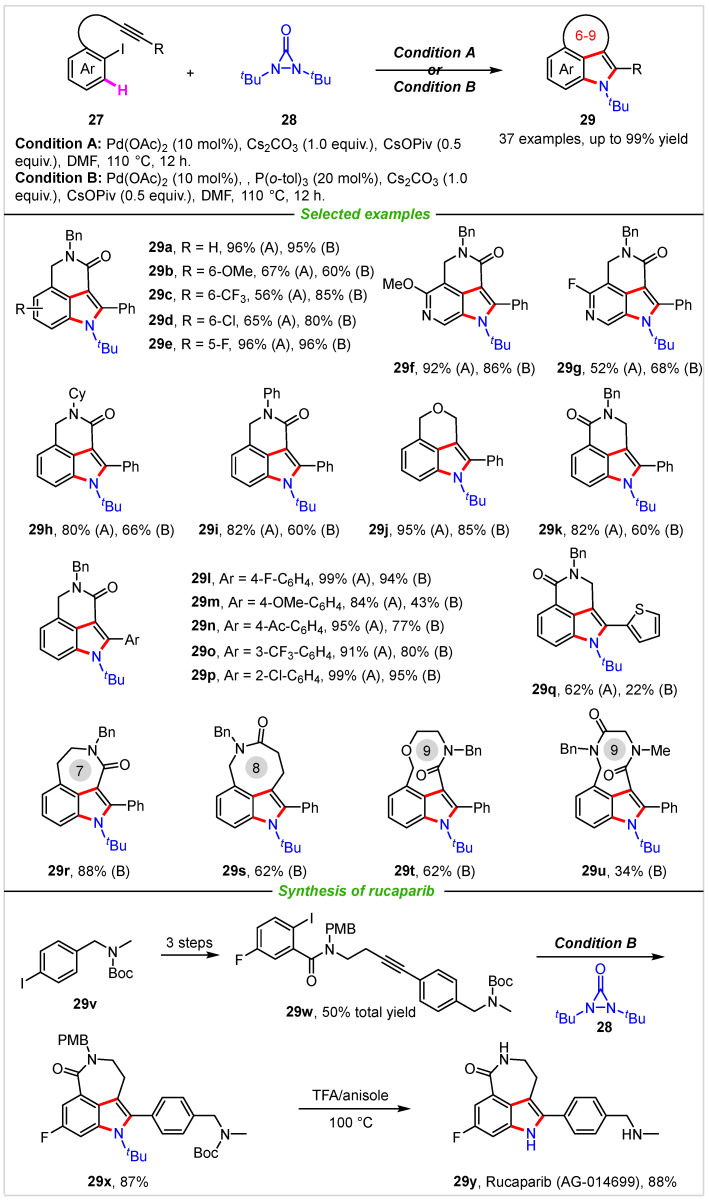
Synthesis of 3,4-fused tricyclic indoles via Pd(0)-catalyzed annulation of alkyne-tethered aryl iodides with diaziridinone.

**Scheme 14:**
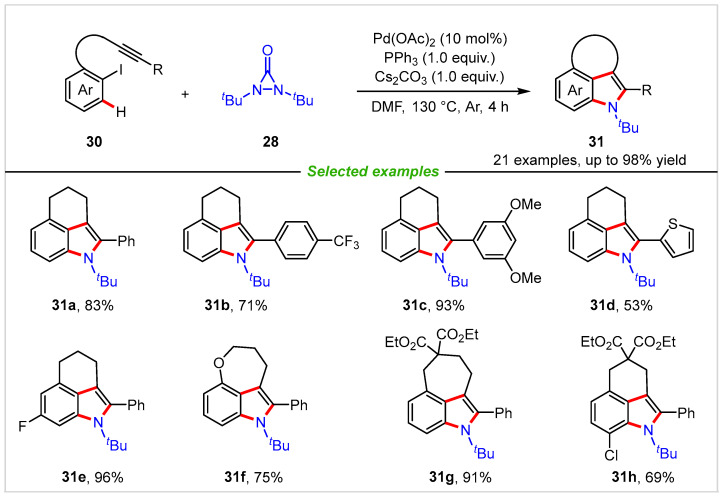
Pd(0)-catalyzed [2 + 2 + 1] annulation of alkyne-tethered aryl iodides with diaziridinone.

Almost simultaneously, the Zhang group, and Yu and Jiang group independently developed an efficient Pd(0)-catalyzed intermolecular annulation of *ortho*-alkyne tethered aryl iodides with *N,N*-di-*tert*-butyldiaziridinone **28** for the synthesis of 3,4-fused tricyclic indoles (Scheme 13 and Scheme 14).

Zhang and co-workers [51] accessed a range of 3,4-fused tricyclic indoles **29,** in moderate to excellent yields with good functional group tolerance, including various ring sizes and various linkers (Scheme 13). Initially, the authors reported a catalyst system without ligand; most of them reacted well and delivered the desired tricyclic indole products in good yields. However, some substrates give lower yields or dramatically shut down the reaction. When an electron-rich and bulky monodentate phosphine ligand P(*o*-tol)_3_ was added, it showed a better performance than no ligand, such as (**29g**,**29r**–**29u**). Notably, the reaction was highlighted by the synthesis of poly(ADP-ribose) polymerase-1 (PARP-1) Inhibitor rucaparib (AG-014699) in 87% yield, for the treatment of advanced solid tumors [52,53].

In Yu and Jiang group’s work [54], they mainly reported carbon-tethered 3,4-fused tricyclic indoles in moderate to excellent yields with wide functional group tolerance (Scheme 14). However, only one example involved oxygen-tethered 3,4-fused tricyclic indole, **31f**, 75% yield. Unfortunately, attempts with alkyl-substituted and unsubstituted alkynes were unsuccessful.

**Scheme 15:**
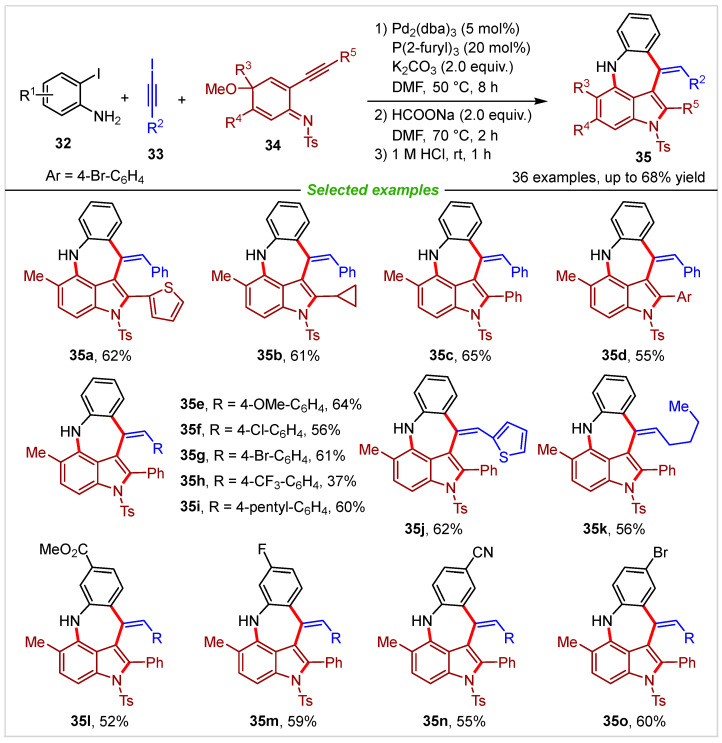
Synthesis of 3,4-fused tricyclic indoles via palladium-catalyzed intermolecular [3 + 3 + 1] annulation of *ortho*-iodoanilines with alkynyl iodides and cyclohexadienimines.

In the same year, Fan, He, and co-workers [55] successfully developed a novel three-component reaction for rapid assembly of 3,4-fused tricyclic indole derivatives from *ortho*-iodoanilines **32**, alkynyl iodides **33**, and cyclohexadienimines **34** using Pd(0) as a tandem catalysis (Scheme 15). Notably, the three starting materials are cheap and readily available. From the insight of mechanics, firstly, this tandem process involves a Pd-catalyzed intermolecular coupling/cyclization/Michael addition and a new chemical bond at the position of C3 and C4 was built. Then, a seven-membered C3, C4 bridge was erected through intramolecular cyclization with Pd(0)-catalysis, and the desired tricyclic indoles were obtained via rearomatization with 1 M aqueous hydrochloric acid. A series of *ortho*-iodoanilines could be tolerated well and delivered desired products (**35l**–**35o**) in acceptable yields. Moreover, iodoalkynes with different substituents such as also reacted smoothly under the standard reaction conditions yielding the desired products (aryl **35e**–**35i**, heterocycle **35j**, and alkyl **35k**). Notably, HCO_2_Na plays a critical role in this transformation, which is used to trigger the necessary transformation in the catalytic cycles.

**Scheme 16:**
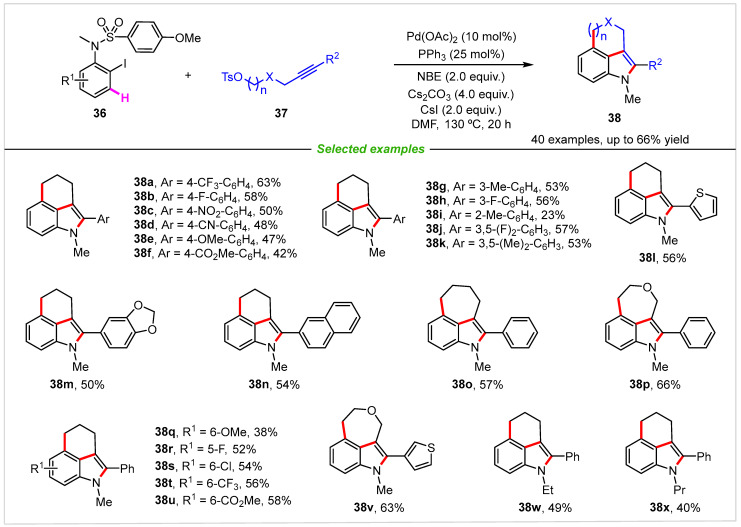
Synthesis of 3,4-fused tricyclic indole derivatives via a N–S bond cleavage strategy with palladium and norbornene in a co-catalysis.

Palladium/norbornene (NBE) chemistry, also known as the Catellani reaction, is widely used in natural product synthesis, complex molecules, functional materials, and other fields, which involves a Pd(0)-catalyzed bifunctionalization of aryl halides with various electrophilic reagents and terminal reagents in the presence of NBE [56]. Seminal works by Catellani, Lautens, Dong, Gu, Liang, Luan, and other groups [57] revealed that a series of polysubstituted aromatics, carbocyclic, and heterocyclic compounds could be obtained by Pd/NBE-catalyzed difunctionalization of aryl halides. In 2021, Zhang, Liang, Li, Quan, and co-workers [58] disclosed an elegant method for the synthesis of 3,4-fused tricyclic indoles via a N–S bond cleavage strategy in one step using palladium and NBE in a co-catalysis (Scheme 16). After extensively screening different amino protecting groups, *para*-methoxy benzenesulfonyl was used as the optimal protecting group, giving the desired product in moderate yield. Regarding the substrate scope, both six- and seven-membered rings could be formed with carbon and oxygen as a linker (**38a**–**38x**). Aryl iodides bearing various groups were tolerated well, yielding the desired products (methoxy **38q**, fluoro **38r**, chloro **38s**, trifluoromethyl **38t**, and ester **38q**). Aryl alkynes bearing different groups in any site of the benzene ring also reacted smoothly under standard conditions to give the expected products **38a**–**38k**. Unfortunately, alkyl alkynes and terminal alkynes were not suitable coupling partners for this reaction. Notably, corresponding products can also be obtained by replacing the methyl with ethyl (**38w**) and propyl (**38x**). Finally, the proposed mechanism was studied by control experiments and density functional theory (DFT) calculations.

**Scheme 17:**
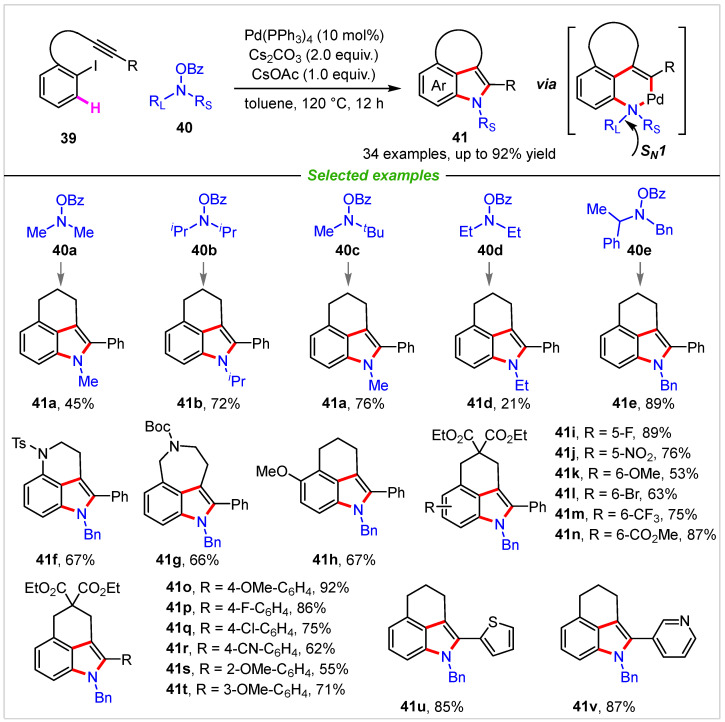
Synthesis of 3,4-fused tricyclic indoles through selective C(sp^3^)-N bond cleavage of tertiary hydroxylamines with the loss of a larger alkyl group.

Very recently, Luan and co-workers [59] reported an efficient Pd(0)-catalyzed [2 + 2 + 1] annulation of *ortho*-alkyne-tethered aryl iodides **39** with tertiary hydroxylamines **40** for the synthesis of 3,4-fused tricyclic indoles **41**, which are widely present in various natural products and medicine molecules (Scheme 17). Compared to their previous work [45], tertiary hydroxylamines were used as nitrogen sources, which are more accessible and more stable. To obtain the optimal reaction conditions, various tertiary hydroxylamines were tested to harness the modest synthetic efficiency at this stage. Using *N*-benzyl-*N*-methyl-benzyl substituent hydroxylamine (**40e**) could greatly improve the performance of titled conversion, affording the desired tricyclic indole (**41e**) in 89% yield through C-N bond cleavage of tertiary hydroxylamine with loss of a larger alkyl group. A series of aryl iodides could be well-tolerated and delivered tricyclic indoles (**41h**–**41n**) in 63–89% yields. Furthermore, with respect to the alkyne terminus, the aromatic rings bearing various substituents also reacted well, affording the desired products (methoxy **41o**, **41s** and **41t**, fluoro **41p**, chloro **41q**, and cyano **41r**) in good to excellent yields. Particularly, heteroarenes such as pyridine and thiophene reacted with (**40e**) to afford (**41v**) and (**41u**) in 87% and 85% yields, respectively. Notably, this novel method was highlighted by a large-scale reaction and the removal of an alkyl group. After systematic mechanistic studies, the selective C(sp^3^)-N bond cleavage mechanism is inclined to the S_N_1 pathway.

## 4. Application in Total Synthesis of Representative Natural Products

**Scheme 18:**
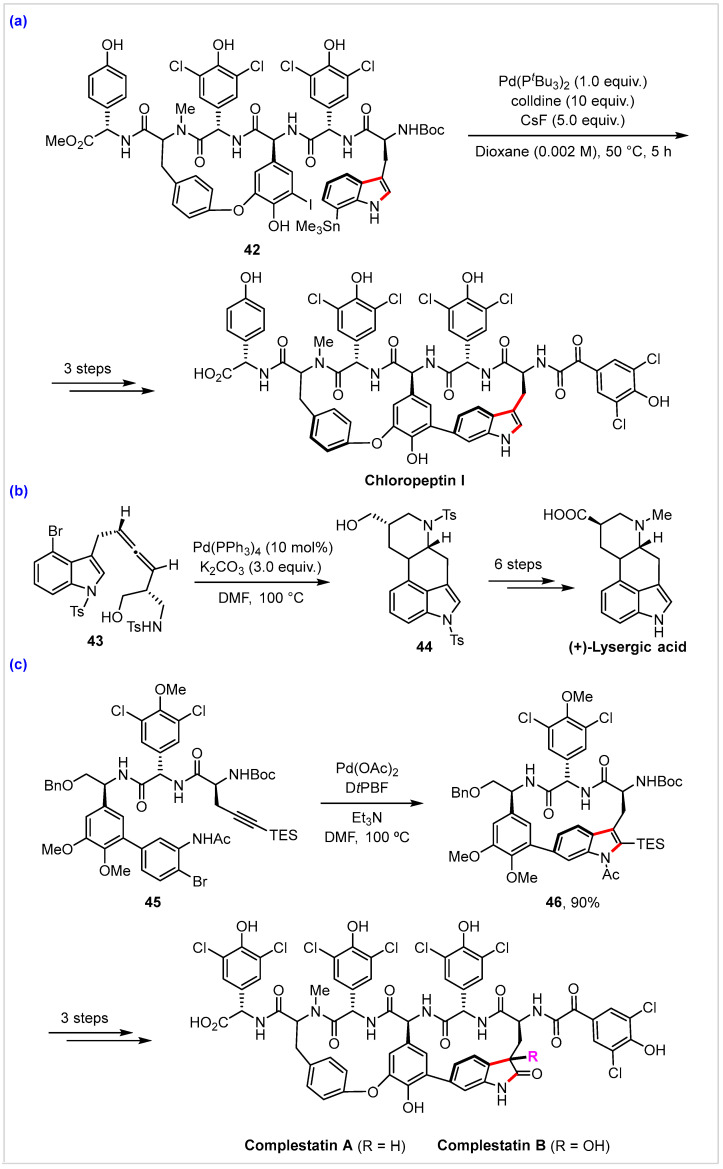
Total synthesis of (**a**) chloropeptin I, (**b**) (+)-lysergic acid, and (**c**) complestatin A and B.

As mentioned above, tricyclic indoles are widely embedded in a number of biologically active natural products. Thus, tremendous efforts have been devoted to the synthesis of some representative natural products using these established methods. Chloropeptin I is an anti-HIV agent obtained from *Streptomyces* sp. WK-3419 [60,61,62]. In 2003, Hoveyda, Snapper, and co-workers [63] reported the first stereoselective total synthesis of anti-HIV agent chloropeptin I by involving palladium-promoted oxidative addition, C-H cleavage, and reductive elimination (Scheme 18a). Undoubtedly, this example is practical and efficient, although a 1.0 equivalent of Pd(P*^t^*Bu_3_)_2_ was used. Lysergic acid is one of important members of ergot alkaloids [64], which oriented its unique tetracyclic skeleton containing a [*cd*]-fused indole, and the asymmetric total syntheses was first achieved in 2004 by the Szántay group [65]. Five years later, Fukuyama and co-workers [66,67] also reported a novel method for synthesis of this natural product. In 2011, Ohno, Fujii, and co-workers [68] realized another method for the asymmetric syntheses of lysergic acid (Scheme 18b). Tricyclic indole **44** was obtained in 80% yield via Pd(0)-catalyzed domino cyclization of the allene-tethered indole **43**. As the analogs of chloropeptin, complestatin A and B were obtained by the fermentation of *Streptomyces* sp. MA7234; Singh and co-workers [69] disclosed it as inhibitors of HIV-1 integrase in 2001, and elucidated its structure and partial stereochemistry. In 2011, Boger and co-workers [70] devised an elegant strategy to achieve the synthesis of complestatin A and B via intramolecular Lacork indole cyclization (Scheme 18c).

**Scheme 19:**
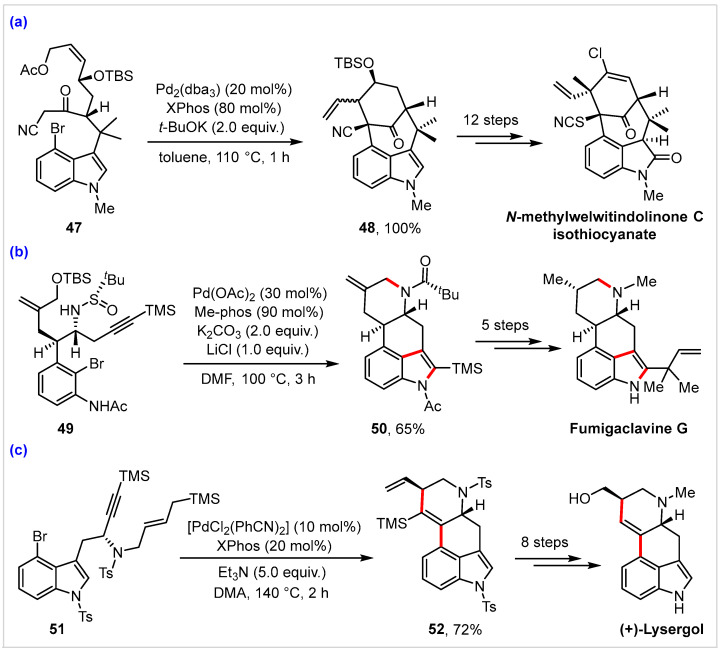
Total synthesis of (**a**) *N*-methylwelwitindolinone C isothiocyanate, (**b**) fumigaclavine G, and (**c**) (+)-lysergol.

*N*-methylwelwitindolinone C isothiocyanate is a member of welwitindolinone alkaloids, which is isolated from the blue-green algae *Hapalosiphon wetwitschii*, *Westiella intricata*, *Fischerella muscicola*, and *Fischerella major* [71,72]. In 2015, Hatakeyama and co-workers [73] developed a Pd(0)-catalyzed tandem enolate strategy for the total synthesis of *N*-methylwelwitindolinone C isothiocyanate (Scheme 19a). Notably, a tetracyclic indole skeleton **48** could be obtained from **47** in 100% yields with 2:1 stereoselectivity. In 2017, Jia and co-workers [74] demonstrate a Pd/Me-phos-catalyzed intramolecular Larock indole annulation/Tsuji-Trost allylation cascade reaction to assemble tetracyclic indole product fumigaclavine G in one step with good yields (Scheme 19b). Moreover, festuclavine, pyroclavine, costaclavine, *epi*-costaclavine, pibocin A, 9-deacetoxyfumigaclavine C, and dihydrosetoclavine, a member of the ergot alkaloids family, were also totally synthesized with this established method. In the same year, Werz and co-workers [75] reported the facile enantioselective total synthesis of (+)-lysergol via Pd(0)-catalyzed *anti*-carbopalladation/Heck cascade process and further eight steps with an overall yield of 13% (Scheme 19c).

**Scheme 20:**
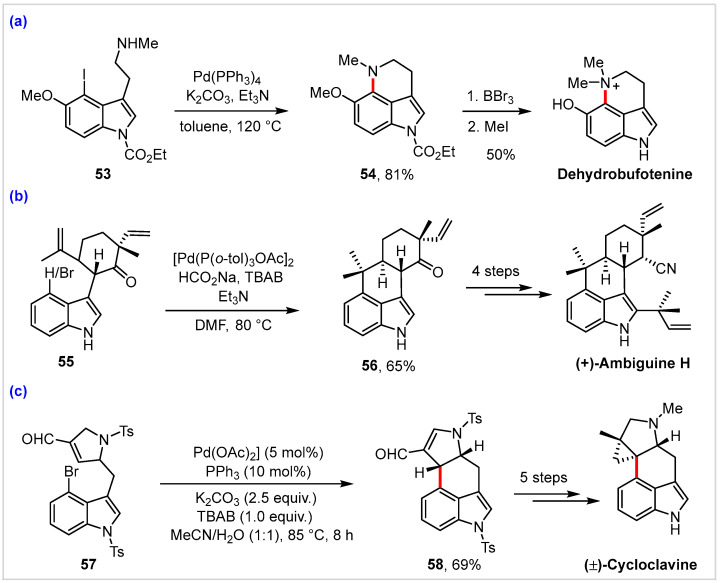
Synthesis of (**a**) dehydrobufotenine, (**b**) (+)-ambiguine H, and (**c**) (±)-cycloclavine.

Dehydrobufotenine, a member of the 1,3,4,5-tetrahydropyrrolo [4,3,2-*de*]quinoline ring system natural products, was originally isolated from the parotid glands of the toad Bufo marinus [76]. In 1996, based on their previous studies about the C–N bond forming reaction, Buchwald and co-workers [77] successfully developed a palladium-catalyzed efficient protocol for the total synthesis of dehydrobufotenine from readily available substrate **53** via intramolecular Buchwald-Hartwig amination/demethylation/methylation reactions. In this manner, dehydrobufotenine was synthesized via a three-step transformation from **53**, affording the desired product in 40.5% overall yield (Scheme 20a). As a member of marine natural products, Ambiguine H produced from cyanobacteria have shown excellent biological activities such as antibacterial and anticancer. Considering the importance of the scaffold in pharmaceutical and natural products, numerous efficient methods have been developed to afford it. In 2007, Baran and co-workers [78] developed a practical method for the enantioselective total syntheses of tetracyclic indole product (+)-ambiguine H via a palladium-catalyzed intramolecular Heck annulation followed by further transformation (Scheme 20b). Compared to previous methods, this method featured no need of a protecting group, shorter steps, and single enantiomers. Moreover, this method is also applicable for synthesis of hapalindole U, welwitindolinone A, and fischerindole I from commercially available materials, where fewer steps were involved in these routes. Cycloclavine, a member of clavine-type alkaloids, was first isolated in 1969 from the seeds of the African morning glory [79]. Over the past few years, only two racemic examples have been reported for the synthesis of cycloclavine [80,81]. So, synthesis of cycloclavine molecules using an efficient strategy is necessary. In 2013, Cao and co-workers [82] reported an efficient and concise strategy for the formal synthesis of (±)-cycloclavine with an intramolecular Heck reaction and iron-catalyzed aza-Cope-Mannich cyclization as key steps.

## 5. Conclusions

3,n-fused tricyclic indoles are widespread in various natural products, drug molecules, and material sciences. Given the importance of these skeletons and complexity of their structures, assembling these skeletons has presented numerous interests and challenges to synthetic chemists. Over the past decades, considerable efforts have been devoted to develop synthetic methods for producing diversified 3,n-fused tricyclic indole derivatives with transition metals as effective catalysts. In particular, Pd(0) complexes stand out as prominent catalysts in the annulation of alkyne-tethered *ortho*-haloanilines or aryl iodides with various amination agents, and have received much attention. In this review, we mainly summarized the recent progress in the synthesis of 3,n-fused tricyclic indoles with a Pd catalyst, and its applications in the total synthesis of representative natural products. By providing an overview of palladium catalysis to access various 3,n-fused tricyclic indole derivatives, we believe that this review will inspire the further developments of novel strategies to achieve tricyclic indoles by using mild and sustainable conditions, extended the space of these tricyclic indole molecules, and potentially given access to new compounds.

## Data Availability

Not applicable.

## Abbreviations

D*t*BPF	1,1′-Bis(di-tert-butylphosphino)ferrocene
TES	triethylsilyl
TMS	trimethylsilyl
DMF	*N*,*N*-dimethylformamide
DMA	*N,N*-dimethylacetamide
DCM	dichloromethane
THF	tetrahydrofuran
DMSO	dimethyl sulfoxide
TFA	trifluoroacetic acid
dba	dibenzylideneacetone
*m*-CPBA	*m*-chloroperbenzoic acid
Me-phos	dicyclohexyl(2′-methyl-[1,1′-biphenyl]-2-yl)phosphane
DPPP	1,3-bis(diphenylphosphaneyl)propane
TBAB	tetrabutylammonium bromide
SN1	unimolecular nucleophilic substitution
Boc	*tert*-butoxycarbonyl
SCN	isothiocyanato
DDQ	4,5-dichloro-3,6-dioxocyclohexa-1,4-diene-1,2-dicarbonitrile
OTBS	*tert*-butyldimethylsilyloxy
PCC	pyridinium chlorochromate
PMB	*para*-methoxlphenyl
DFT	density functional theory
*o*-PBA	*ortho*-phenyl benzoic acid
DPPBz	1,2-Bis(diphenylphosphino)benzene
NBE	norbornene
NMR	nuclear magnetic resonance
HRMS	high resolution mass spectrometry
Xphos	dicyclohexyl(2′,4′,6′-triisopropyl-[1,1′-biphenyl]-2-yl)phosphane
